# Improved prediction of symptomatic type 1 diabetes using a luciferase‐based assay to measure (pro)insulin autoantibodies

**DOI:** 10.1111/dme.70277

**Published:** 2026-04-23

**Authors:** Rebecca C. Wyatt, Cristina Brigatti, Sian L. Grace, Claire L. Williams, Ilaria Marzinotto, Ben T. Gillard, Elena Bazzigaluppi, Deborah Shoemark, Michela A.‐M. Chandler, Peter Achenbach, Lorenzo Piemonti, BOX Study Group, Isabel Wilson, Rachel Aitken, Ilana Kelland, Clare Megson, Chitrabhanu Ballav, Atanu Dutta, Michelle Russell‐Taylor, Rachel Besser, James Bursell, Shanthi Chandran, Sejal Patel, Anne Smith, Manohara Kenchaiah, Gomathi Margabanthu, Foteini Kavvoura, Chandan Yaliwal, AWAF, Rachel Aitken, Olivia Pearce, Sarah Stollery, Elinor Balch, Hanah Batholomew, Zhara Hashmi, Kathleen M. Gillespie, Vito Lampasona, Alistair J. K. Williams, Anna E. Long

**Affiliations:** ^1^ University of Bristol Medical School Bristol UK; ^2^ IRCCS Ospedale San Raffaele Milan Italy; ^3^ University of Bristol School of Biochemistry Bristol UK; ^4^ Helmholtz Zentrum Munchen Institute of Diabetes Research Munich Bavaria Germany; ^5^ Klinikum Rechts der Isar der Technischen Universitat Munchen Munich Bavaria Germany

**Keywords:** antibody affinity, autoimmunity, diabetes mellitus, insulin antibodies, luciferase, predictive value of tests, type 1

## Abstract

**Introduction:**

Insulin autoantibodies (IAA) are key predictors of type 1 diabetes, particularly in young children. Micro‐radiobinding assays (RBA) are the gold standard for IAA measurement but have limitations. We assessed whether a luciferase immunoprecipitation system (LIPS) assay improved diabetes risk assessment.

**Methods:**

To validate LIPS compared with RBA, samples from people with new‐onset type 1 diabetes (*n* = 150) and first‐degree relatives (FDRs) (*n* = 619), of whom 91 had developed diabetes during follow‐up, were used. This cross‐sectional observational data was analysed using the area under the receiver operator characteristic curve and cox‐proportional hazard models.

**Results:**

In new‐onset diabetes, RBA and LIPS showed 88% agreement in IAA status. Positive IAA LIPS was more common in 89 FDRs with high‐moderate affinity IAA (61%) compared with 22 FDRs with low‐affinity IAA (18%) (*p* < 0.001). In FDRs positive for multiple other islet autoantibodies, 20‐year diabetes risk was 80% for those positive compared with 30% for those negative for IAA by LIPS (*p* = 0.013). IAA LIPS added to diabetes risk independently of status/level of IAA by RBA, other autoantibodies and sampling age (*p* < 0.001).

**Conclusion:**

The IAA LIPS low‐blood‐volume, high‐throughput technique identifies more individuals with the highest risk of diabetes. The ability to identify high‐affinity IAA makes LIPS an ideal method for future clinical trials and population screening strategies to predict the risk of diabetes.

**What's New?**

- We compared the performance of luciferase immunoprecipitation system (LIPS) assays measuring IAA with the classic micro‐radiobinding assay (RBA).
- The LIPS methods are sensitive and specific; they more often identify high‐/moderate‐affinity IAA compared with RBA. LIPS IAA stratify the risk of progression independently of RBA status/level, other islet autoantibodies, RBA titre and age at sample.
- These LIPS assays combine practical advantages with better diagnostic performance that could improve feasibility and identification of high‐risk individuals for recruitment to a clinical trial or general population screening.

## 1 INTRODUCTION

Insulin autoantibodies (IAA) are a key predictor of type 1 diabetes. Often the first marker of islet autoimmunity, these autoantibodies are frequently detected prior to disease onset and are particularly prevalent in young children who progress to disease rapidly.^1^, ^2^ At diagnosis, levels of IAA show an inverse correlation with age up to ~12 years, after which they are less common.^3^, ^4^ Critically, IAA positivity is regularly used as a selection criterion and/or outcome measure to target potential therapeutics at individuals with the highest risk of developing diabetes.^5^

High‐affinity IAA are associated with a young age at seroconversion, development of multiple islet autoantibodies and/or progression to type 1 diabetes in relatives of people with type 1 diabetes and schoolchildren.^6^, ^7^ High‐affinity autoantibodies bind strongly to the A‐chain residues 8–13 of insulin and proinsulin.^6^ Low‐affinity autoantibodies, however, often react to C‐terminal residues of the B‐chain, do not bind to proinsulin and are not associated with disease.^6^

Historically, radiobinding assays (RBA) were the gold standard for IAA detection.^8^ Large natural history studies including TEDDY and TrialNet both measure IAA using the RBA. These measurements have been used in models to examine progression rate to type 1 diabetes.^9^, ^10^ The combination of GADA and IAA was associated with relatively lower risk in TrialNet with a 17.5% risk within 5 years. It is possible that this low risk is partly driven by the lower‐risk IAA‐positive individuals detected by RBA.

We optimised a novel, fluid‐phase luciferase immunoprecipitation system (LIPS) assay to measure IAA.^11^ Preliminary results (*n* = 136 relatives) suggested that measuring IAA by LIPS using luciferase‐tagged proinsulin or insulin constructs better discriminated diabetes risk compared with RBA using ^125^I‐insulin.^11^

The aim of the current study, therefore, was to use a large, unique cohort of new‐onset patients and first‐degree relatives (FDRs), in order to (1) validate the LIPS technique for comparison with the RBA and (2) compare the prediction of diabetes in detail with other factors that stratify risk in a unique population with up to 37 years follow‐up.

## 2 METHODS

### 2.1 Population

Serum samples with sufficient volume were identified from participants of the Bart's‐Oxford (BOX) type 1 diabetes family study which has been recruiting since 1985.^12^ Autoantibodies to 65‐kDa glutamate decarboxylase (GADA), islet antigen‐2 (IA–2A) and insulin were previously measured by RBA.^13^ Autoantibodies to Zinc Transporter‐8 (ZnT8A) were measured by RBA in all sera included in this study.^13^ Relatives were followed for diabetes development by annual questionnaire. The BOX study was approved by the South Central – Oxford C. National Research Ethics Committee. Participants provided informed, written consent and the study was performed according to the principles of the Declaration of Helsinki.

#### 2.1.1 Evaluation of the sensitivity of LIPS assays

**FIGURE 1:**
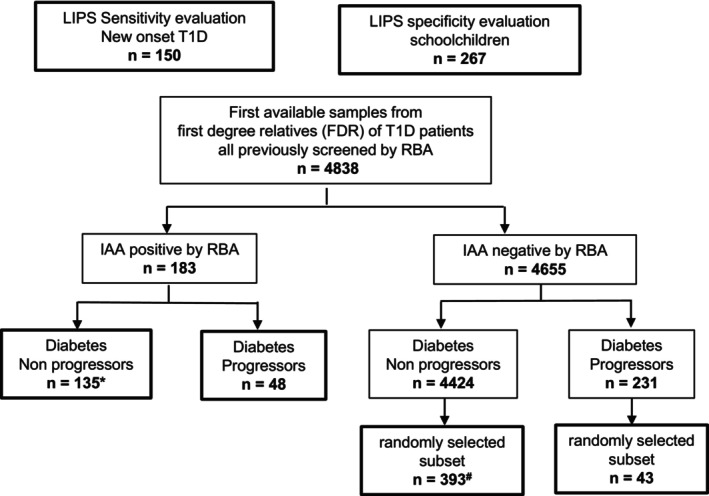
Testing strategy flow diagram. * 1 excluded from survival analysis #8 excluded from survival analysis due to lack of follow‐up data. Final groups for analysis shown in boxes with bold outline, including a total of 436 IAA negative by RBA.

From a collection of 423 samples from people with new‐onset type 1 diabetes,^13^ taken within 2 weeks of diagnosis (median duration 0 day, range −61 to 13), 150 with sufficient serum were randomly selected (Table 1 and Figure 1).

**TABLE 1:** Population characteristics.

	People with type 1 diabetes (*n* = 150)	IAA‐positive relatives (*n* = 183)		IAA‐Negative relatives (*n* = 436)	
		Progressor (*n* = 48)	Non‐progressor (*n* = 135)	Progressor (*n* = 43)	Non‐progressor (*n* = 393)
No. males (%)	81 (54%)	22 (46%)	62 (46%)	32 (74%)	187 (48%)
Median age, years (range)	10.4 (1.3–20.7)	27.8 (2.1–54.8)	31.4 (2.6–60.2)	39.3 (7.8–50.8)	30.7 (0.4–55.9)
Median age at diagnosis, years (range)	10.4 (1.3–20.7)	37.3 (3.3–68.0)	‐	51.7 (9.4–72.0)	‐
Median diabetes duration, days (range)/Median follow‐up, years	0 (−61–13)	8.4 (1.1–28.9)	18.6 (0–36.1)	14.5 (1.6–26.5)	19.5 (0–36.8)
No follow‐up, *n*	‐	0	1	0	8
≥2 Islet autoantibodies (IAA excluded), *n* (%)	124 (83)	23 (48)	11 (8)	2 (5)	3 (1)
≥2 Islet autoantibodies (IAA included), *n* (%)	133 (89)	33 (69)	29 (21)	2 (5)	3 (1)
RBA IAA Positive, *n* (%)	109 (73)	48 (100)	135 (100)	0 (0)	0 (0)
GADA Positive, *n* (%)	122 (81)	31 (65)	26 (19)	3 (7)	17 (4)
IA‐2A Positive, *n* (%)	123 (82)	15 (31)	8 (6)	2 (5)	4 (1)
ZnT8A Positive, *n* (%)	121 (81)	23 (48)	11 (8)	3 (7)	2 (1)

*Note*: Progressor: First‐degree relative known to have developed diabetes during follow‐up. Non‐progressor: First‐degree relative who has not developed diabetes during follow‐up.

#### 2.1.2 Evaluation of the specificity of LIPS assays

The first available sample collected from 4838 relatives had been screened previously for IAA by RBA (Figure 1). We measured IAA by LIPS assay in all 183 FDRs originally found IAA positive by RBA, including 48 subjects who progressed to diabetes during follow‐up and 29 with autoantibodies to ≥ 2 islet antigens (including IAA) (Table 1 and Figure 1).

To interrogate assay specificity, we also analysed a subset of 436 FDRs from the remaining 4655 FDRs who had screened IAA negative by RBA. This subset was randomly selected except for enrichment with 43 of 231 IAA negative FDRs who developed diabetes (Table 1 and Figure 1) and an additional 3 who had multiple islet autoantibodies and did not develop diabetes. Development of diabetes for BOX relatives not tested for IAA LIPS is shown in Figure S1.

### 2.2 Recombinant luciferase‐tagged (pro)insulin antigen production

Recombinant proinsulin and insulin (‘(pro)insulin’ encompasses both proinsulin and insulin) antigens were expressed as described previously^11^ (Figure S2). Two different luciferase‐tagged (pro)insulin constructs were utilised with Nluc incorporated at the C‐terminus of the proinsulin or insulin B‐chain: bNluc‐proinsulin and bNluc‐insulin, respectively. The protein yield was up to 1.2 × 10^10^ LU/μl for luciferase‐tagged (pro)insulins in a total volume of 30 mL of cell culture supernatant. This was aliquoted, stored at −70°C and syringe‐filtered (PVDF 0.22 μm) before use.

### 2.3 IAA LIPS assay

For both LIPS assays, the luciferase‐tagged antigens were diluted in 50 mM Tris, pH 8 with 0.5% v/v Tween‐20 (TBT) to a concentration of 9.95 × 10^6^–1.05 × 10^7^ LU per 5 μL. Sera were pipetted (1 μL, 4 replicates) into a 96‐round deep‐well plate (Beckman Coulter, Brea, CA, USA), incubated with 5 μL diluted antigen for 24 h at 4°C, with and without unlabelled insulin (ULI) (Actrapid®, Novo Nordisk, Bagsværd, Denmark) added at a final concentration of 3.5 × 10^−7^mol/L. Immunocomplexes were precipitated using 2.5 μL glycine‐blocked Protein A Sepharose 4 fast flow and 1.25 μL ethanolamine‐blocked Protein G sepharose (GE Healthcare Life Sciences, Chicago, IL, USA) (washed 4 times in TBT) for 1 h with shaking (~700 rpm). Precipitates were washed 5 times with TBT, transferred to a 96‐well OptiPlate™ (Perkin‐Elmer, Waltham, MA, USA) and excess buffer was removed by aspiration. Nano‐Glo® substrate (40 μL, Promega) was injected into each well immediately before counting in a LB 960 microplate Centro XS3 luminometer (Berthold Technologies). In the 2020 IASP workshop, the adjusted sensitivity at 95% was 62% for the RBA and 64% for both bNluc‐proinsulin and bNluc‐insulin LIPS assays (Lab 116).^14^

### 2.4 Autoantibody quantification, thresholds and affinity measurements

Autoantibody levels are reported in local units, interpolated from a standard curve comprising pooled patient sera diluted in healthy control sera.

Positive thresholds for IAA using ^125^I‐insulin, bNluc‐proinsulin and bNluc‐insulin were set using the 97.5th percentile of 267 healthy schoolchildren (9–13 years old, 139 (52%) male). These were 0.2, 0.26 and 0.24 units for the RBA, bNluc‐proinsulin and bNluc‐insulin, respectively.

RBA IAA affinity data were available for 111 (61%) of the 183 IAA‐positive FDRs. Results were classed as high‐moderate or low affinity, based on previously described methods.^15^

### 2.5 Statistical analysis

Categorical and continuous variables were compared using McNemar's test with Yate's correction, Fisher's exact test, Wilcoxon signed‐rank tests or Spearman's correlation, as appropriate. Concordance of IAA‐positive/negative scores across assays were expressed as average pairwise percent agreement.^16^ Assay performance was analysed using the area under the Receiver Operator Characteristics curve (ROC‐AUC) and the partial ROC‐AUC at 95% specificity (pAUC95).^17^ The ROC‐AUC or pAUC95 were compared between assays using the bootstrap method calculated using the pROC package in R.^18^ Mantel‐Cox log‐rank test was used to compare progression to diabetes (outcome) by IAA status (exposure). Cox regression models included predictors RBA IAA (status/level), autoantibody number, age at sample using IBM SPSS Statistics (version 28.0.1.1(15)). The 5‐year risk (rapid progression) and 20‐year risk (median follow‐up of all BOX relatives) are reported with 95% CI. The RBA IAA level was categorised at 0.6 units (median for IAA‐positive FDRs). Autoantibody number was for GADA, IA‐2A and ZnT8A, unless stated. Sampling age was categorised at 24 years old (the nadir between the parents' and siblings' ages, Figure S3). Analyses were performed using the R software version 4.0.3 unless otherwise specified. Participants with missing data were excluded.

## 3 RESULTS

### 3.1 The sensitivity and specificity of the IAA LIPS assays were comparable to the IAA RBA

#### 3.1.1 New‐onset type 1 diabetes and healthy school children

**FIGURE 2:**
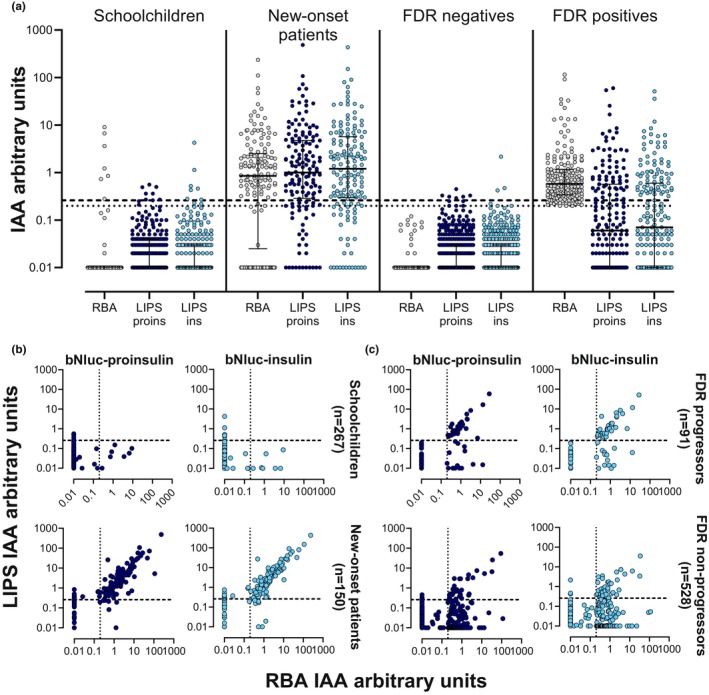
(p)IAA levels in individuals with different diabetes status. Plot of RBA, Nluc‐PIAA and bNluc‐IAA levels in 267 school children (without diabetes at time of testing), 150 people with new‐onset type 1 diabetes, 436 RBA IAA negative first‐degree relatives (FDR negatives), and 183 RBA IAA positive first‐degree relatives (FDR positives). (a) Dashed line is Nluc‐(P)IAA threshold (0.26 units), dotted line is RBA IAA threshold (0.2 units). (b) Plot of Nluc‐PIAA and bNluc‐IAA levels against IAA levels measured by RBA in 267 schoolchildren and 150 people with new‐onset type 1 diabetes. Correlation of Nluc‐PIAA and bNluc‐IAA with RBA IAA was very good (*r* = 0.89, *p* < 0.0001 and *r* = 0.90, *p* < 0.0001, respectively). (c) Plot of RBA IAA, Nluc‐PIAA and bNluc‐IAA levels in first‐degree relatives who did (*n* = 91, *r* = 0.65, *p* < 0.0001 and *r* = 0.71, *p* < 0.001, respectively) and did not (*n* = 528, *r* = 0.32, *p* < 0.0001 and *r* = 0.36, *p* < 0.0001, respectively) progress to diabetes. Grey is RBA IAA, light blue is Nluc‐PIAA bNluc‐IAA.

Of 150 people with new‐onset type 1 diabetes, 108 (72%) were IAA positive by RBA and 114 (76%) were positive by the bNluc‐proinsulin and 114 (76%) were positive by the bNluc‐insulin LIPS assays (Figure 2a), and 107 (71%) were positive by both LIPS constructs. In these people, IAA positive and negative scores by RBA showed 88% agreement between either LIPS assay. In 267 schoolchildren, IAA positive and negative scores showed 95% percent agreement between RBA and LIPS assays (*n* = 249 (94%) negative by all assays).

In new‐onset type 1 diabetes, IAA levels were highly correlated between RBA and the two LIPS assays (bNluc‐proinsulin *r* = 0.883 and bNluc‐insulin LIPS *r* = 0.897, *p* < 0.0001 for both) (Figure 2b). In schoolchildren, IAA levels measured by RBA and bNluc‐proinsulin (*r* = 0.121, *p* = 0.049) or bNluc‐insulin LIPS were poorly correlated (*r* = 0.045, *p* = 0.465) (Figure 2b). Comparison of the LIPS assays is shown in Figure S4.

The ROC‐AUC was 0.86 (95% CI 0.82–0.99) for RBA, compared with 0.93 (0.89–0.96, *p* < 0.001) for bNluc‐proinsulin and 0.92 (0.89–0.96, *p* < 0.001) for bNluc‐insulin LIPS assays. The pAUC95 was 0.028 (0.018–0.036), 0.038 (0.033–0.042) and 0.035 (0.028–0.041), respectively (RBA vs. bNluc‐proinsulin *p* = 0.031, RBA vs. bNluc‐insulin *p* = 0.168, Figure S5).

#### 3.1.2 First‐degree relatives

Of the 619 FDRs, 183 (30%) were IAA positive by RBA and 64 (10%, *p* < 0.0001) by the bNluc‐proinsulin and 71 (11%, *p* < 0.001) by bNluc‐insulin LIPS (Figure 2a), 59 (10%) were positive by both assays. The correlation of IAA levels between the RBA and two LIPS assays was high in FDRs who progressed to diabetes (*n* = 91): bNluc‐proinsulin r = 0.637 (*p* < 0.001) and bNluc‐insulin *r* = 0.750 (*p* < 0.001) (Figure 2c). In 528 FDRs who did not develop diabetes, IAA levels by RBA and bNluc‐proinsulin (*r* = 0.302, *p* < 0.001) or bNluc‐insulin LIPS (*r* = 0.346, *p* < 0.001) were correlated, but less strongly (Figure 2c). There were 3 non‐progressors IAA negative by RBA but positive by bNluc‐insulin; one of these was also positive by bNluc‐proinsulin.

Measurement of affinity by RBA was available for 111 FDRs positive by RBA. Of those who had high‐moderate affinity IAA, 54/89 (61%) were positive by LIPS compared with 4/22 (18%) who had low‐affinity IAA (*p* < 0.001).

Of the 48 FDR progressors who were IAA positive by RBA, 31 (65%) were positive by bNluc‐proinsulin and 32 (66%) by bNluc‐insulin LIPS assay. Of the 135 IAA‐positive non‐progressors positive by RBA, only 43 (31%) were also positive in the LIPS assays.

#### 3.1.3 First‐degree relative progressors positive by IAA RBA but negative by IAA LIPS were older than those positive by both methods

**FIGURE 3:**
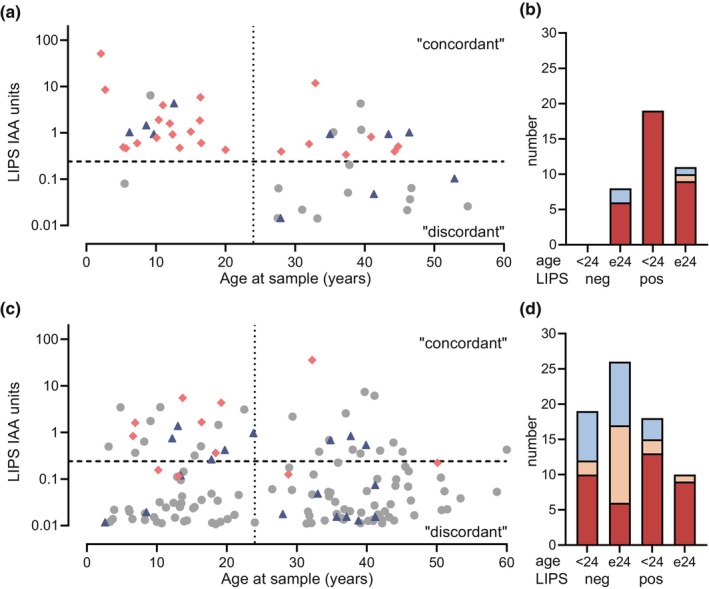
IAA level by age, affinity and other autoantibodies. In relatives who progress to diabetes (a, b). (a) IAA levels of bNluc‐insulin LIPS are plotted for RBA IAA positive progressors (*n* = 48). (b) IAA affinity in progressors (*n* = 38). In relatives who do not progress to diabetes (c, d). (c) IAA levels of bNluc‐insulin LIPS are plotted for RBA IAA positive non‐progressors (*n* = 135). (d) IAA affinity in non‐progressors (*n* = 73). Bar charts subdivided by LIPS‐insulin positive or negative and age at sample 24 years old. In (a, c) colour indicates number of GADA, IA‐2A and/or ZnT8A, grey = none, dark blue = single, pale red = multiple. In (b, d) colour indicates affinity, light blue = low, orange = moderate, dark red = high affinity. In (a, c) vertical dotted line indicates 24 years old (nadir between parents and sibling ages). Horizontal dashed line indicate threshold for LIPS IAA positive (0.24 units).

Due to the similarity between bNluc‐proinsulin and bNluc‐insulin LIPS, bNluc‐insulin alone is discussed in further analysis. Of 48 FDR progressors IAA positive by RBA, 14 (29%) were negative by the bNluc‐insulin LIPS assay (‘discordant progressors’, median follow‐up 8.5y, range 1.2–22.2). Discordance was not related to IAA level but was related to age at sampling. Of 14 discordant progressors, 13 (93%) were sampled above 24 years of age, while only 13/34 (38%) of ‘concordant progressors’ (RBA and bNluc‐insulin positive) were sampled above 24 years of age (*p* = 0.040, Figure 3a). Therefore, all but 1 discordant progressor developed diabetes as adults (median age at diagnosis 52.4 year, range 6.7–67.1). In ROC analysis LIPS discriminated progression better than RBA for younger, but not older FDRs (Figure S6). None of the 23 IAA RBA positive multiple autoantibody (excluding IAA) positive progressors were negative for LIPS (Figure 3a). Of those with affinity data; 6/8 (75%) discordant progressors had high‐moderate affinity IAA compared with 29/30 (97%) concordant progressors (*p* = 0.106, Figure 3b).

#### 3.1.4 First‐degree relative non‐progressors positive by IAA RBA but negative by IAA LIPS had fewer other autoantibodies and lower‐affinity IAA

Of 393 FDR non‐progressors IAA negative by RBA, 3 were bNluc‐insulin positive. Amongst FDR non‐progressors who were RBA IAA positive, 98/135 (73%) were discordant between bNluc‐insulin and RBA and this was not related to IAA level. Of these, 59 (60%) were sampled above 24 years of age compared with 16/37 (43%) concordant FDRs sampled above 24 years of age (*p* = 0.084, Figure 3c). The discordant non‐progressors had fewer other autoantibodies than the concordant non‐progressors (*p* = 0.002, Figure 3c). Of those with affinity data, 29/45 (64%) discordant non‐progressors had high‐moderate affinity compared with 25/28 (89%) of concordant non‐progressors (*p* = 0.027, Figure 3d).

### 3.2 Time‐to‐event analysis in first‐degree relatives

#### 3.2.1 First‐degree relatives positive for IAA by LIPS have an increased risk of diabetes development

**FIGURE 4:**
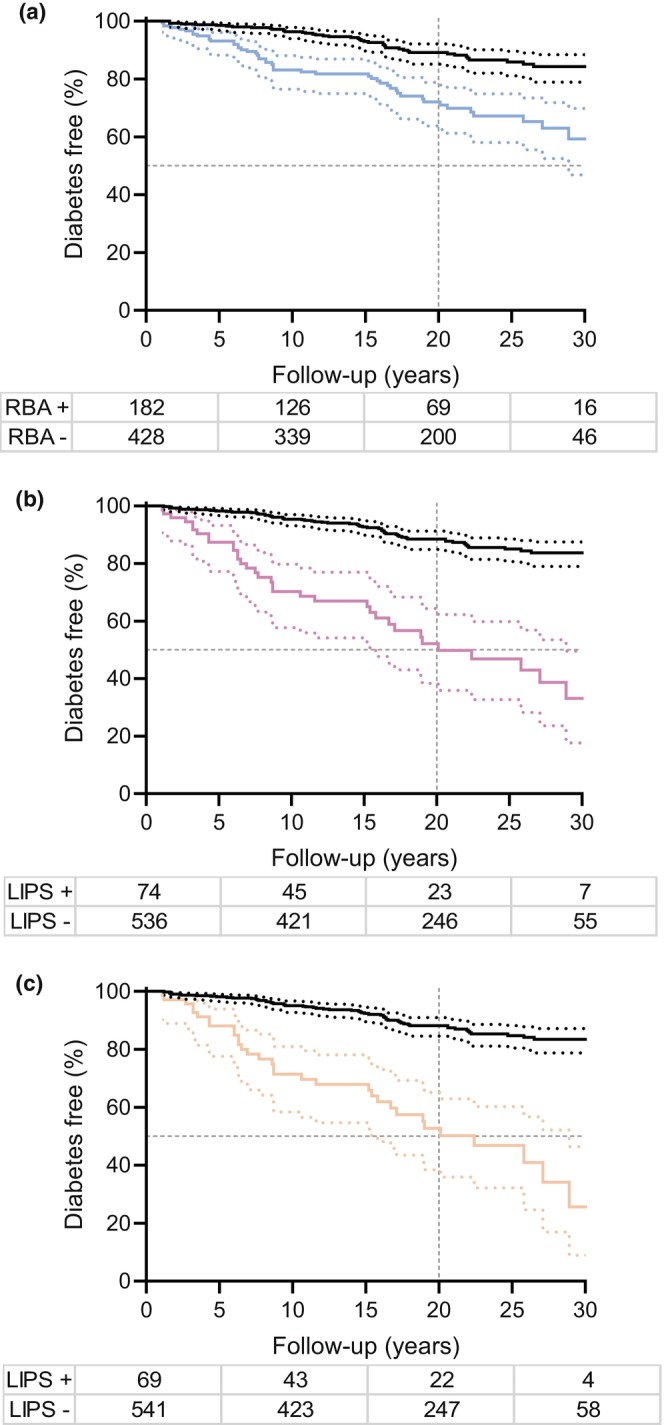
Diabetes free survival (%) in all relatives stratified by assay type. (a) RBA IAA positive (blue). (b) bNluc‐insulin positive (pink). (c) bNluc‐proinsulin positive (orange). Black line negative in each sub‐figure. *p* < 0.0001 for all. 20‐year diabetes risk was 11.3% (95% CI 8.2–15.4) in RBA negative, 29% (22.1–37.5) in RBA positive, 12.3% (9.3–16) in bNluc‐insulin negative, 50.2% (37.8–64.1) in bNluc‐insulin positive, 12.6% (9.6–16.3) in bNluc‐proinsulin negative, 49.8% (37.1–64.1) in bNluc‐proinsulin positive.

Development of diabetes in 610 FDRs was investigated by time‐to‐event analysis (Figure 4). Stage 3 type 1 diabetes at 20 years of follow‐up was less frequent in RBA IAA–positive relatives, 29% (95% CI 22.1–37.5), compared with 50.2% (37.8–64.1, *p* = 0.001) in bNluc‐proinsulin IAA–positive FDRs, and 49.8% (37.1–64.1, *p* = 0.001) in bNluc‐insulin IAA‐positive FDRs. During the first 5 years of follow‐up, the 95% CI for risk of diabetes overlapped between RBA and LIPS (Table S1).

#### 3.2.2 IAA LIPS further stratified risk in relatives positive by RBA, independent of RBA level in those with multiple islet autoantibodies, and across different ages

**FIGURE 5:**
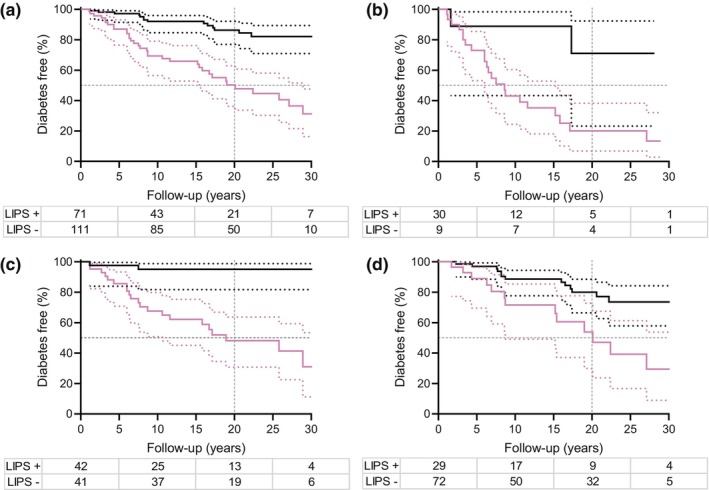
Stratification of risk of diabetes by LIPS bNluc‐insulin in relatives. (a) RBA IAA positive (*p* < 0.0001). (b) with multiple islet autoantibodies (exc. IAA) positive (*p* = 0.0132). (c) under 24 years old at sampling (*p* < 0.0001). (d) over 24 years old at sampling (*p* = 0.0017). bNluc‐insulin positive (pink), bNluc‐insulin negative (black). Shading represent 95% CI. Dotted lines show 50% diabetes at 20 years of follow‐up. *p*‐values from Mantel‐Cox Log‐rank test.

The ability of bNluc‐insulin to further stratify risk in the context of other risk factors was investigated (Table 2). RBA IAA affinity was not included in time‐to‐event analysis because of missing data due to limited sample volume. Within 182 IAA RBA–positive FDRs, bNluc‐insulin stratified risk (*p* < 0.0001, Figure 5a), this was irrespective of IAA titre (data not shown). Multiple islet autoantibody–positive FDRs (*n* = 39) positive by bNluc‐insulin had the highest observed 20‐year risk of diabetes (79.9%, 61.7–93.1), compared with multiple islet autoantibody positive but LIPS IAA–negative FDRs (28.9%, 7.7–76.7) (*p* = 0.0132, Figure 5b). In 571 FDRs with either zero or one other autoantibody (GADA, IA‐2A or ZnT8A), bNluc‐insulin did not stratify risk. In both younger (<24 years old) and older FDRs, bNluc‐insulin stratified risk (Figure 5c,d). Similar results were obtained for bNluc‐proinsulin (data not shown).

**TABLE 2:** Risk of developing diabetes within 20 years of sampling stratified by bNluc‐insulin LIPS.

Group	*n*	5‐year progression (%) (95% CI)		20‐year progression (%) (95% CI)		Overall *p*‐value
		LIPS negative	LIPS positive	LIPS negative	LIPS positive	
RBA IAA positive	182	2.8% (0.9–8.5)	13% (7–23.5)	15.6 (9.2–25.8)	52.2 (39.4–66.3)	<0.0001
RBA IAA <0.6 units	89	1.7% (0.2–11.6)	10% (3.3–27.9)	12.7 (5.8–26.5)	37.4 (22–58.7)	0.0018
RBA IAA ≥0.6 units	93	2% (0.3–13.4)	15.3% (7.2–30.9)	19.7 (9.6–38.2)	66.2 (47.7–83.7)	<0.0001
Aab negative	525	1.9% (1–3.6)	3.8% (0.6–24.3)	10.2 (7.5–13.8)	11.2 (2.7–40.3)	0.8836
sAab positive	46	No cases	No cases	22.6 (9.9–46.4)	52.1 (27.3–81.8)	0.3173
mAab positive	39	11.1% (1.6–56.7)	27% (14.5–46.8)	28.9 (7.7–76.7)	79.9 (61.7–93.1)	0.0132
Under 24 years old at sampling	83	2.4% (0.3–16.1)	14.4% (6.7–29.2)	4.9 (1.3–18.3)	51.8 (36.3–69.2)	<0.0001
Over 24 years old at sampling	101	3% (0.8–11.5)	11% (3.7–30.4)	22.9 (13.4–37.3)	52.9 (32.5–76.3)	0.0017

*Note*: *p*‐values from the Mantel–Cox Log‐rank test. 5‐year and 20‐year risk of diabetes (95% CI).

In Cox regression analysis including IAA RBA status/level (negative, <0.6 units, >= 0.6 units), age (continuous variable) and number of islet autoantibodies (none, single, multiple), addition of bNluc‐insulin or bNluc‐proinsulin status (positive/negative) improved the model (*p* = 0.001 and *p* = 0.003, respectively, Table 3). Age, number of islet autoantibodies and (P)IAA LIPS status all contributed to the model (*p* < 0.005 for all), but IAA RBA level did not (Table 3).

**TABLE 3:** Cox regression analysis of diabetes risk in 595 FDR.

	Model without IAA LIPS			Model with IAA LIPS			Model with PIAA LIPS		
	df	*p*‐value	HR (95% CI)	df	*p*‐value	HR (95% CI)	df	*p*‐value	HR (95% CI)
Age at sample (years)^a^	1	<0.001	1.03 (1.02–1.05)	1	<0.001	1.04 (1.02–1.05)	1	<0.001	1.04 (1.02–1.05)
RBA IAA level	2	0.004	Reference (negative)	2	0.058	Reference	2	0.073	Reference
RBA IAA <0.6 units		0.800	1.08 (0.6–1.95)		0.248	0.66 (0.32–1.34)		0.464	0.78 (0.4–1.52)
RBA IAA ≥0.6 units		0.002	2.34 (1.36–4.03)		0.349	1.38 (0.7–2.73)		0.171	1.57 (0.82–3)
Autoantibody number	2	0.000	Reference (none)	2	0.000	Reference	2	<0.001	Reference
Single autoantibody		0.011	2.32 (1.22–4.43)		0.054	1.93 (0.99–3.75)		0.086	1.82 (0.92–3.59)
Multiple autoantibody		<0.001	9.13 (5.23–15.92)		<0.001	6.28 (3.4–11.59)		<0.001	6.72 (3.64–12.44)
(P)IAA LIPS	Not included			1	0.001	3.33 (1.68–6.58)	1	0.004	3.33 (1.68–6.58)
Model characteristics	5	<0.001	Chi‐square: 162.37	6	<0.001	Chi‐square: 182.16	6	0.003	Chi‐square: 175.41

^a^ This was modelled as a continuous variable. Modelling age as <24 years old vs. ≥24 years old did not impact estimates of HR for the other variables (data not shown).

## 4 DISCUSSION

In this study we established that IAA measured by LIPS predicted stage 3 type 1 diabetes in relatives of people with type 1 diabetes. The LIPS assay is a low‐blood volume, high‐throughput technique. It preserves both the sensitivity and specificity when examined in people with diabetes and healthy controls. In FDRs who did not progress to diabetes, it reduced the number identified as IAA positive by three‐quarters, improving the specificity compared with RBA. The overall risk of diabetes progression was increased in IAA LIPS positive compared with IAA LIPS negative FDRs, particularly in individuals with multiple autoantibodies. LIPS outperformed IAA measured by RBA added to risk independently of age, RBA IAA level and number of other islet autoantibodies.

This study was limited by preselection based on previous RBA IAA data this is discussed, where relevant, below. In addition, individuals were not followed from birth; however, there was a uniquely long follow‐up of participants, above 35 years. This enabled interrogation of the relationship between IAA and diabetes development over a prolonged period in both children and adults. The pathogenesis of type 1 diabetes in adults, who represent more than half of incident cases,^19^, ^20^ may hold clues to mechanisms of autoimmune regulation leading to delayed onset.

In 2020, Jacobson et al. showed that the risk of type 1 diabetes development within 5 years is highly variable in individuals positive for multiple autoantibodies (measured by RBA), which was also inversely associated with age.^10^ The combination of IAA/GADA was the most common among those with 2 autoantibodies; however, it provided the lowest risk of progression compared with other autoantibody pairs. About one third of FDRs positive for IAA by RBA who progressed to diabetes were IAA negative by LIPS, suggesting reduced sensitivity. However, all but one of these 14 FDRs were adults when tested and diagnosed, and only 3 had additional islet autoantibodies. Single autoantibody–positive adults appear to be a lower‐risk group according to a recent TrialNet analysis.^21^ The difference in sensitivity may be offset by an improvement in specificity; nonetheless, some caution may be necessary in employing the IAA LIPS assay in adults. Overall, our data showed that IAA LIPS more effectively discriminated risk of diabetes development.

Multiple other platforms have emerged for measurement of islet autoantibodies including electrochemiluminescence (ECL) and Antibody Detection by Agglutination‐PCR (ADAP). Analysis of IASP IAA workshops (2018 and 2020) suggests LIPS, RBA and ECL are strongly performing methods. Recent analysis in TrialNet relatives previously screened for islet autoantibodies by RBA compared to RBA, ECL, LIPS and ADAP for IAA. This suggested that LIPS had the highest sensitivity for prediction of stage 3 type 1 diabetes within 5 years within individuals with multiple autoantibodies. Additional studies would be required to validate this observation. If confirmed, IAA LIPS could be pivotal in accurately identifying high‐risk rapid progressors for inclusion in clinical trials as distinct from those progressing slowly.^22^, ^23^

Moreover, additional characteristics of autoantibodies, such as affinity, influence risk of progression. For example, single positivity for GADA or IAA is associated with much higher diabetes risk when defined by high‐affinity electrochemiluminescence compared with RBA.^24^ IAA measured by LIPS were also more frequently high to moderate affinity. The LIPS assay competes binding of labelled insulin with a lower concentration of unlabelled insulin (3.5 × 10^−7^ mol/L) compared with the RBA (4.0 × 10^−5^ mol/L). This would be expected to reduce identification of the lowest affinity autoantibodies. The positioning of the Nluc next to the B‐chain (Figure S2) may also obscure less disease‐relevant lower affinity epitopes.^6^ Potentially explaining the additional value of LIPS when included in multivariable analysis of risk where affinity could not be included. LIPS is potentially a quicker and cheaper alternative to affinity measurement.

Compared with RBAs, LIPS offers a range of practical advantages including reduced regulatory demands and ease of set up within laboratories already equipped to perform RBA. The assay previously described^11^ has been improved further by halving the volume of serum and other key reagents required and by reducing the concentration of unlabelled insulin used to competitively displace IAA. These modifications have both improved the cost effectiveness of the technique and increased IAA measurement specificity. The LIPS method reduces the total serum requirement for IAA measurement to only 4 μL. This compares favourably with the 20 μL required for the RBA^8^ and 15 μL (measurement of multiple autoantibodies) by the ECL technique.^25^ Today, capillary blood testing is increasing in popularity, facilitating general population screening and at‐home collection of samples.^26^, ^27^ Low‐volume assays are key to allow for multiple islet autoantibody testing and to gain maximum information from each sample. As a straightforward method of autoantibody screening, LIPS offers many advantages in facilitating widespread screening, though further work assessing its performance in non‐relatives is required.

Overall, LIPS is a sensitive and specific, low‐cost, high‐throughput assay requiring minimal serum volumes for IAA measurement. The accessibility of this method and its ability to discriminate between progression rates makes this a viable alternative to RBAs with the potential to enhance identification of high‐risk individuals for therapy.

## CONFLICT OF INTEREST STATEMENT

The authors declare that they have no known competing financial interests or personal relationships that could have appeared to influence the work reported in this paper.

## Supporting information


**Figure S1:** Diabetes Free survival in the whole BOX cohort for those tested or not tested for IAA LIPS. (a) Autoantibody negative relatives (progressors were selectively over sampled). (b) Single autoantibody positive relatives (including single IAA RBA positives and IAA‐ other antibody positive (blue text in table)). (c) IAA RBA pos with at least one other autoantibody (all tested by LIPS). (d) IAA RBA positive + multiple other autoantibody positive relatives (this includes IAA positive + at least 2 other autoantibodies and IAA negative + at least 2 other autoantibodies (blue text in table)). Red line – tested by LIPS; Black line – not tested for LIPS. Dotted lines represent 50% risk and 20 years follow‐up.
**Figure S2:** Luciferase tagged (pro) insulin constructs.
**Figure S3:** Age at sampling of relatives included in this study. This included 361 parents and 258 siblings of the proband. We used histograms to identify age 24 years as the nadir/mid‐point between the youngest parent and the oldest sibling, with 4 parents <24 years old and 4 siblings ≥24 years old. The first available sample on entry to BOX was analysed where available. Entry to BOX for relatives was at the time of proband's diagnosis with diabetes.
**Figure S4:** (P)IAA levels in individuals with different diabetes status. Plot of Nluc‐PIAA against bNluc‐IAA levels in 267 schoolchildren and 150 people with new‐onset type 1 diabetes. Spearman's rank correlation of Nluc‐PIAA with bNluc‐IAA was *r* = 0.178 (*p* = 0.003) in schoolchildren, *r* = 0.941 (*p* < 0.001) in people with new‐onset type 1 diabetes, *r* = 0.799 (*p* < 0.001) in first‐degree relatives who did progress to diabetes and 0.364 (*p* < 0.001) in relatives who did not progress to diabetes.
**Figure 5:** ROC curve analysis of people with diabetes and schoolchildren without diabetes. Receiver Operator Characteristic (ROC) curves (a, b) on different scales and IAA positivity based on lab‐defined thresholds (c). In 150 people with type 1 diabetes and 267 healthy schoolchildren. Grey – RBA, dark blue – LIPS‐ProIns, light blue – LIPS‐Ins. (a) Grey shaded area pAUC95. The pAUC95 was 0.028 (0.018–0.036), 0.038 (0.033–0.042) and 0.035 (0.028–0.041), respectively (RBA vs. bNluc‐proinsulin *p* = 0.031, RBA vs. bNluc‐insulin *p* = 0.168).
**Figure S6:** ROC curve analysis of relatives of people with diabetes. (a) ROC curve for relatives under 24 years old at sampling. (b) Percentage of relatives under 24 years at sampling positive for IAA with each method. (c) ROC curve for relatives over 24 years old at sampling. (d) Percentage of relatives over 24 years old at sampling, positive for IAA with each method. Grey – RBA, dark blue – ProIns, light blue – Ins. (a) Grey shaded area pAUC95. Under 24, pAUC95 for RBA 0.004 (95% CI 0–0.013), for ProIns LIPS 0.017 (95% CI 0.006–0.031), for Ins LIPS 0.014 (95% CI 0.006–0.027), *p* = 0.029 (Ins LIPS vs. RBA). Over 24, pAUC95 for RBA 0.002 (95% CI 0–0.004), for ProIns 0.005 (95% CI 0.002–0.009), for Ins LIPS 0.005 (95% 0.002–0.009), *p* = 0.072 (Ins LIPS vs. RBA). Diabetes is self‐reported. In the group sampled over 24 years old only 8 of 66 progressors reported type 1 diabetes, however of those who reported a different diabetes diagnosis 9 had evidence of islet autoantibodies before diabetes diagnosis 2 had multiple islet autoantibodies (1 with and 1 without IAA), 5 had IAA by RBA + 1 other islet autoantibody and 2 had 1 autoantibody (not IAA).
**Table S1:** Test characteristics in relatives who developed (*n* = 91) or did not develop (*n* = 528) clinical type 1 diabetes. * defined by progression to diabetes.
