# Supplementary material for: Improved prediction of symptomatic type 1 diabetes using a luciferase‐based assay to measure (pro)insulin autoantibodies
Source: Diabet Med. 2026 Apr 23;43(8):e70277. doi: 10.1111/dme.70277 (PMC13380342; doi:10.1111/dme.70277)
Supplement: Supplementary file 1 — Figure S1: Diabetes Free survival in the whole BOX cohort for those tested or not tested for IAA LIPS. (a) Autoantibody negative relatives (progressors were selectively over sampled). (b) Single autoantibody positive relatives (including single IAA RBA positives and IAA‐ other antibody positive (blue text in table)). (c) IAA RBA pos with at least one other autoantibody (all tested by LIPS). (d) IAA RBA positive + multiple other autoantibody positive relatives (this includes IAA positive + at least 2 other autoantibodies and IAA negative + at least 2 other autoantibodies (blue text in table)). Red line – tested by LIPS; Black line – not tested for LIPS. Dotted lines represent 50% risk and 20 years follow‐up. Figure S2: Luciferase tagged (pro) insulin constructs. Figure S3: Age at sampling of relatives included in this study. This included 361 parents and 258 siblings of the proband. We used histograms to identify age 24 years as the nadir/mid‐point between the youngest parent and the oldest sibling, with 4 parents <24 years old and 4 siblings ≥24 years old. The first available sample on entry to BOX was analysed where available. Entry to BOX for relatives was at the time of proband's diagnosis with diabetes. Figure S4: (P)IAA levels in individuals with different diabetes status. Plot of Nluc‐PIAA against bNluc‐IAA levels in 267 schoolchildren and 150 people with new‐onset type 1 diabetes. Spearman's rank correlation of Nluc‐PIAA with bNluc‐IAA was r = 0.178 (p = 0.003) in schoolchildren, r = 0.941 (p < 0.001) in people with new‐onset type 1 diabetes, r = 0.799 (p < 0.001) in first‐degree relatives who did progress to diabetes and 0.364 (p < 0.001) in relatives who did not progress to diabetes. Figure 5: ROC curve analysis of people with diabetes and schoolchildren without diabetes. Receiver Operator Characteristic (ROC) curves (a, b) on different scales and IAA positivity based on lab‐defined thresholds (c). In 150 people with type 1 diabetes and 267 healthy schoolch [file DME-43-e70277-s001.pptx]

## Slide 1
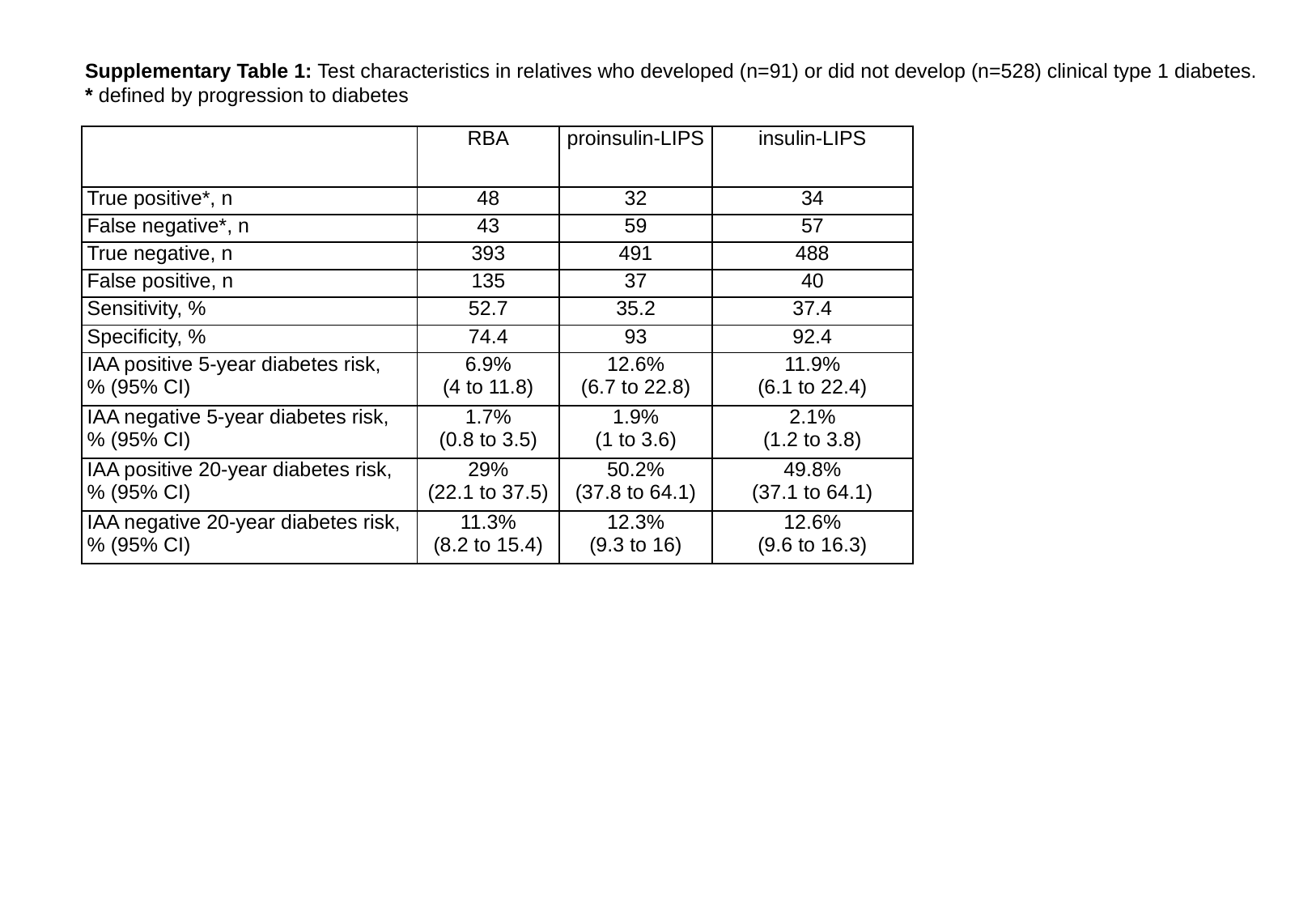

Supplementary Table 1: Test characteristics in relatives who developed (n=91) or did not develop (n=528) clinical type 1 diabetes.
* defined by progression to diabetes
| | RBA | proinsulin-LIPS | insulin-LIPS |
| --- | --- | --- | --- |
| True positive\*, n | 48 | 32 | 34 |
| False negative\*, n | 43 | 59 | 57 |
| True negative, n | 393 | 491 | 488 |
| False positive, n | 135 | 37 | 40 |
| Sensitivity, % | 52.7 | 35.2 | 37.4 |
| Specificity, % | 74.4 | 93 | 92.4 |
| IAA positive 5-year diabetes risk, % (95% CI) | 6.9% (4 to 11.8) | 12.6% (6.7 to 22.8) | 11.9% (6.1 to 22.4) |
| IAA negative 5-year diabetes risk, % (95% CI) | 1.7% (0.8 to 3.5) | 1.9% (1 to 3.6) | 2.1% (1.2 to 3.8) |
| IAA positive 20-year diabetes risk, % (95% CI) | 29% (22.1 to 37.5) | 50.2% (37.8 to 64.1) | 49.8% (37.1 to 64.1) |
| IAA negative 20-year diabetes risk, % (95% CI) | 11.3% (8.2 to 15.4) | 12.3% (9.3 to 16) | 12.6% (9.6 to 16.3) |

## Slide 2
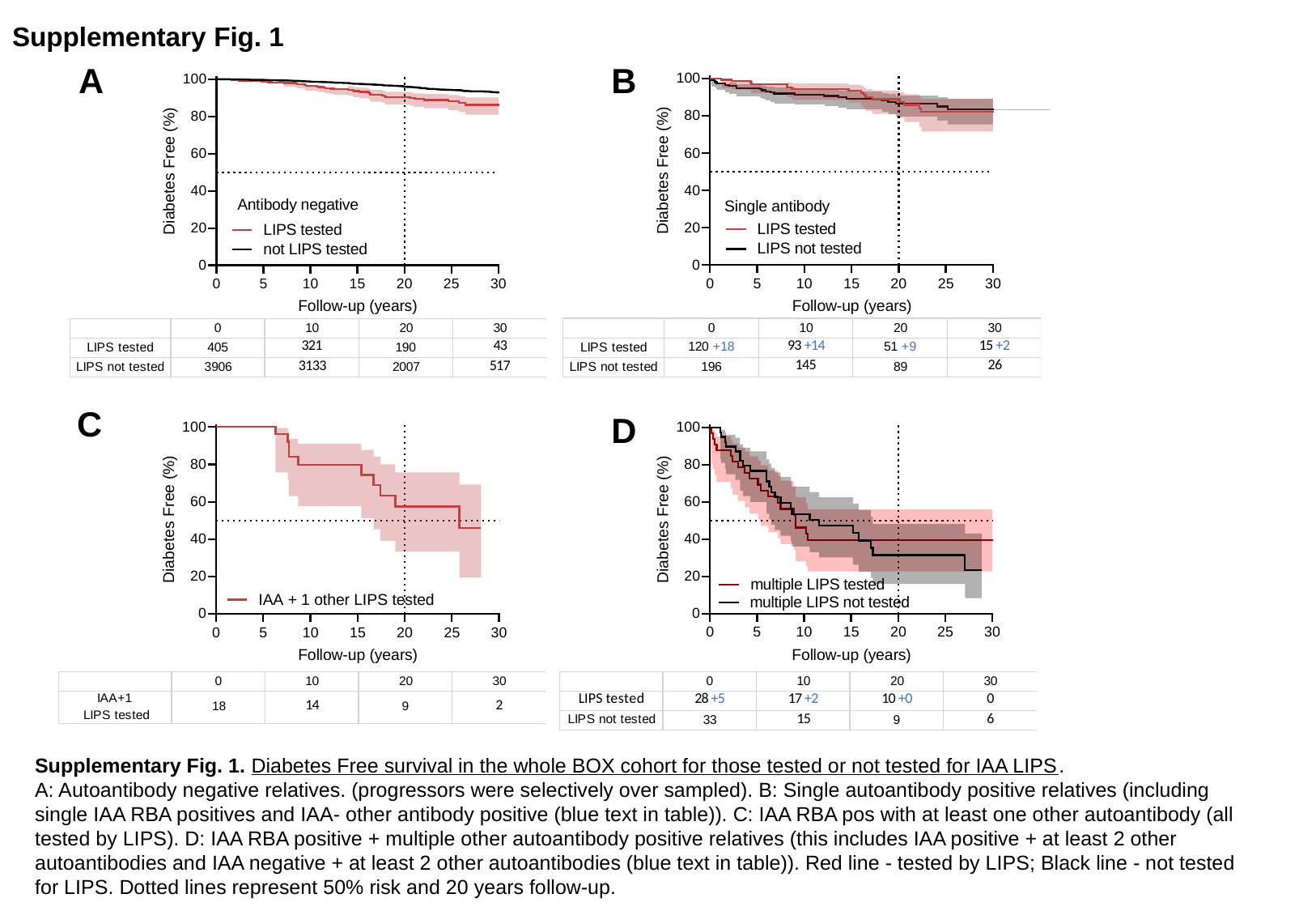

# Supplementary Fig. 1
Supplementary Fig. 1. Diabetes Free survival in the whole BOX cohort for those tested or not tested for IAA LIPS.
A: Autoantibody negative relatives. (progressors were selectively over sampled). B: Single autoantibody positive relatives (including single IAA RBA positives and IAA- other antibody positive (blue text in table)). C: IAA RBA pos with at least one other autoantibody (all tested by LIPS). D: IAA RBA positive + multiple other autoantibody positive relatives (this includes IAA positive + at least 2 other autoantibodies and IAA negative + at least 2 other autoantibodies (blue text in table)). Red line - tested by LIPS; Black line - not tested for LIPS. Dotted lines represent 50% risk and 20 years follow-up.

## Slide 3
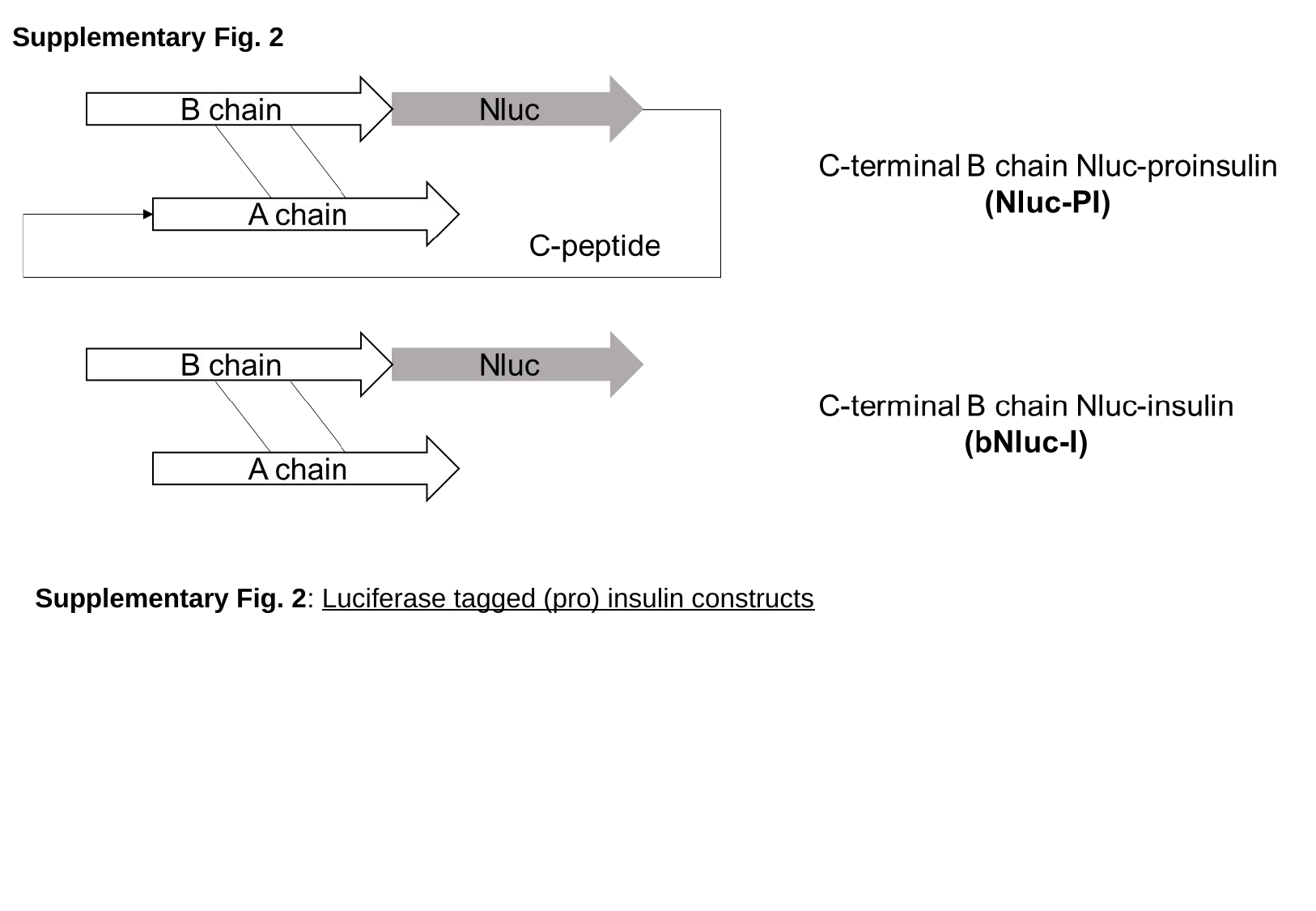

# Supplementary Fig. 2
Supplementary Fig. 2: Luciferase tagged (pro) insulin constructs

## Slide 4
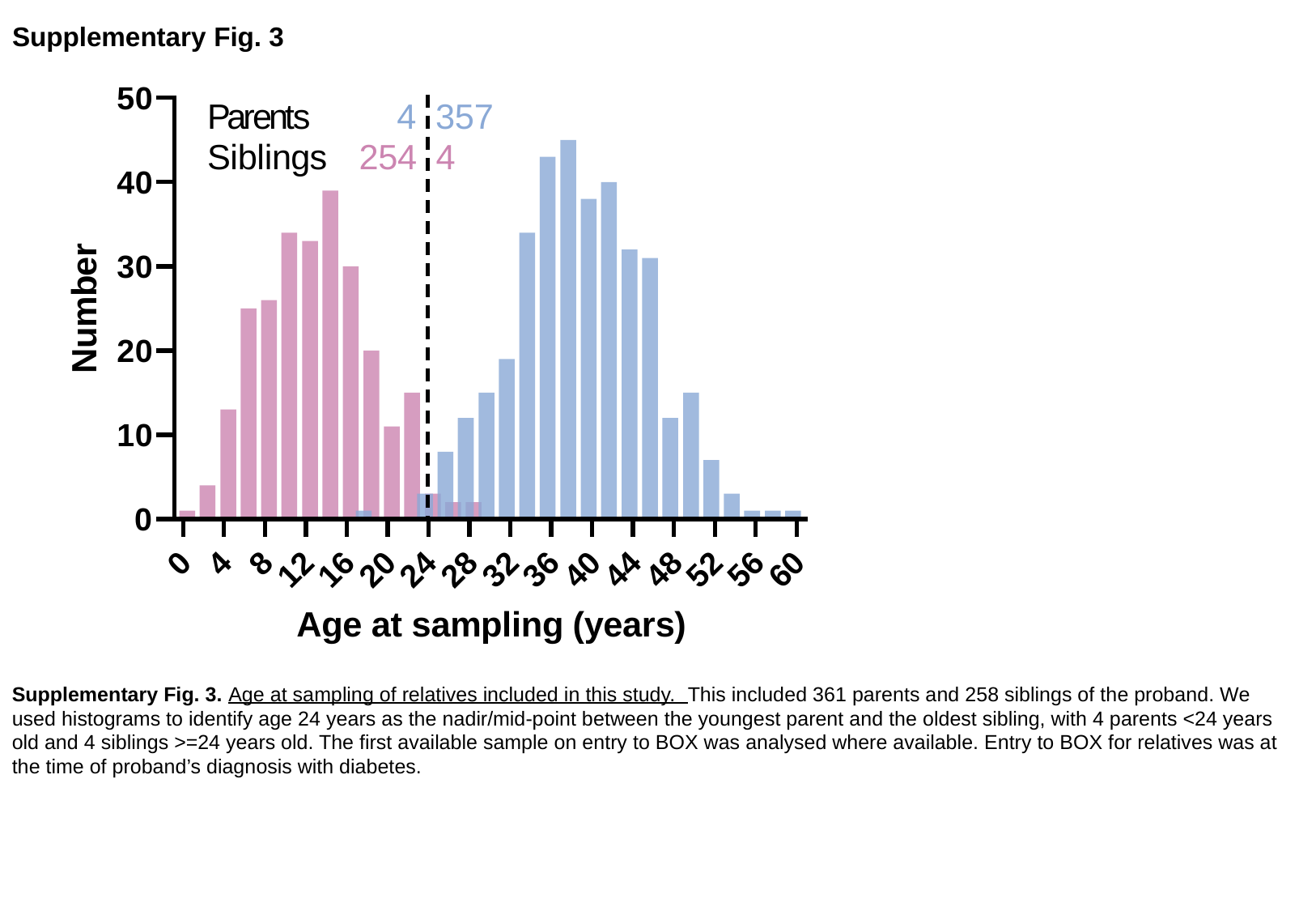

# Supplementary Fig. 3
Supplementary Fig. 3. Age at sampling of relatives included in this study. This included 361 parents and 258 siblings of the proband. We used histograms to identify age 24 years as the nadir/mid-point between the youngest parent and the oldest sibling, with 4 parents <24 years old and 4 siblings >=24 years old. The first available sample on entry to BOX was analysed where available. Entry to BOX for relatives was at the time of proband’s diagnosis with diabetes.

## Slide 5
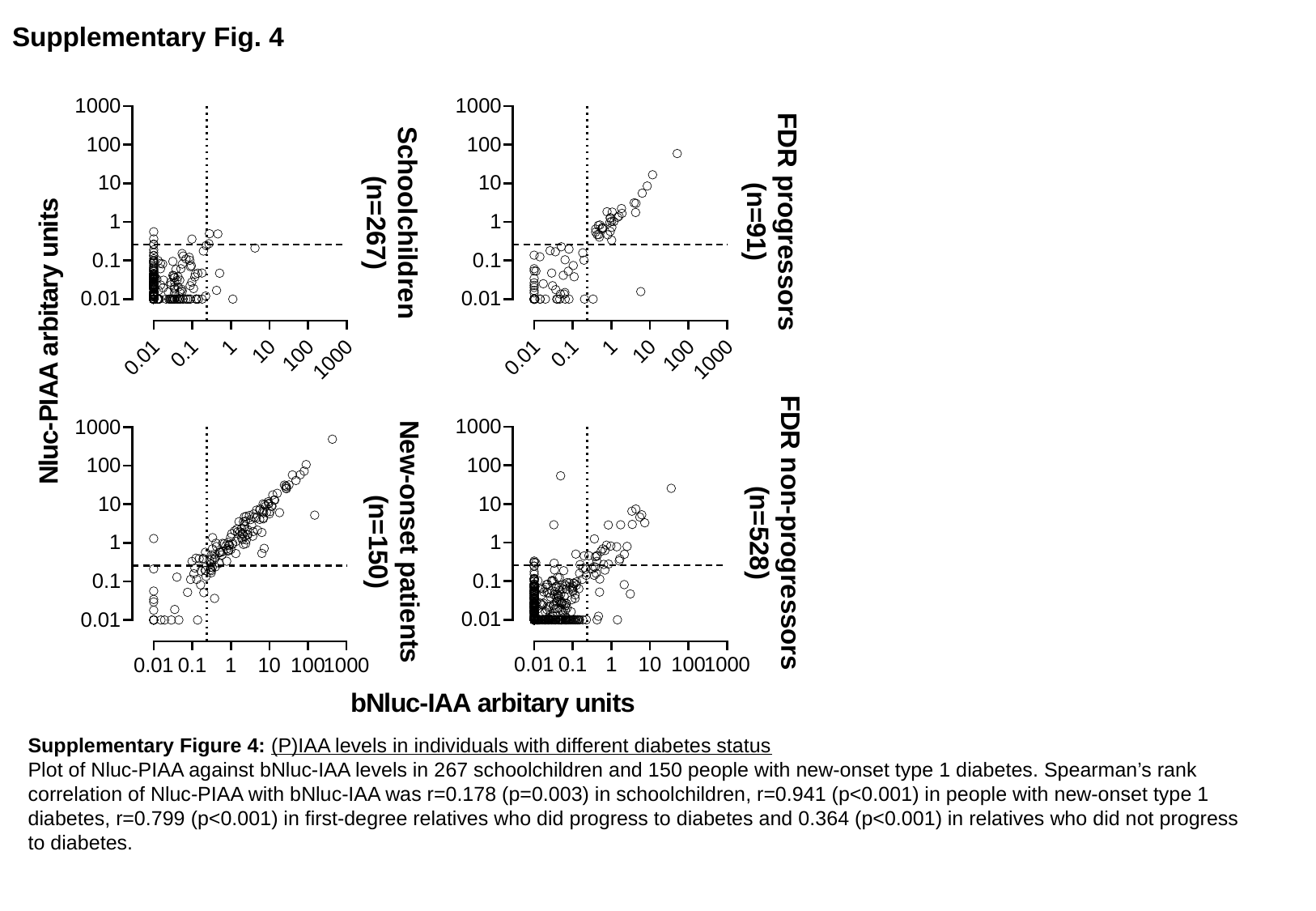

Supplementary Fig. 4
Supplementary Figure 4: (P)IAA levels in individuals with different diabetes status
Plot of Nluc-PIAA against bNluc-IAA levels in 267 schoolchildren and 150 people with new-onset type 1 diabetes. Spearman’s rank correlation of Nluc-PIAA with bNluc-IAA was r=0.178 (p=0.003) in schoolchildren, r=0.941 (p<0.001) in people with new-onset type 1 diabetes, r=0.799 (p<0.001) in first-degree relatives who did progress to diabetes and 0.364 (p<0.001) in relatives who did not progress to diabetes.

## Slide 6
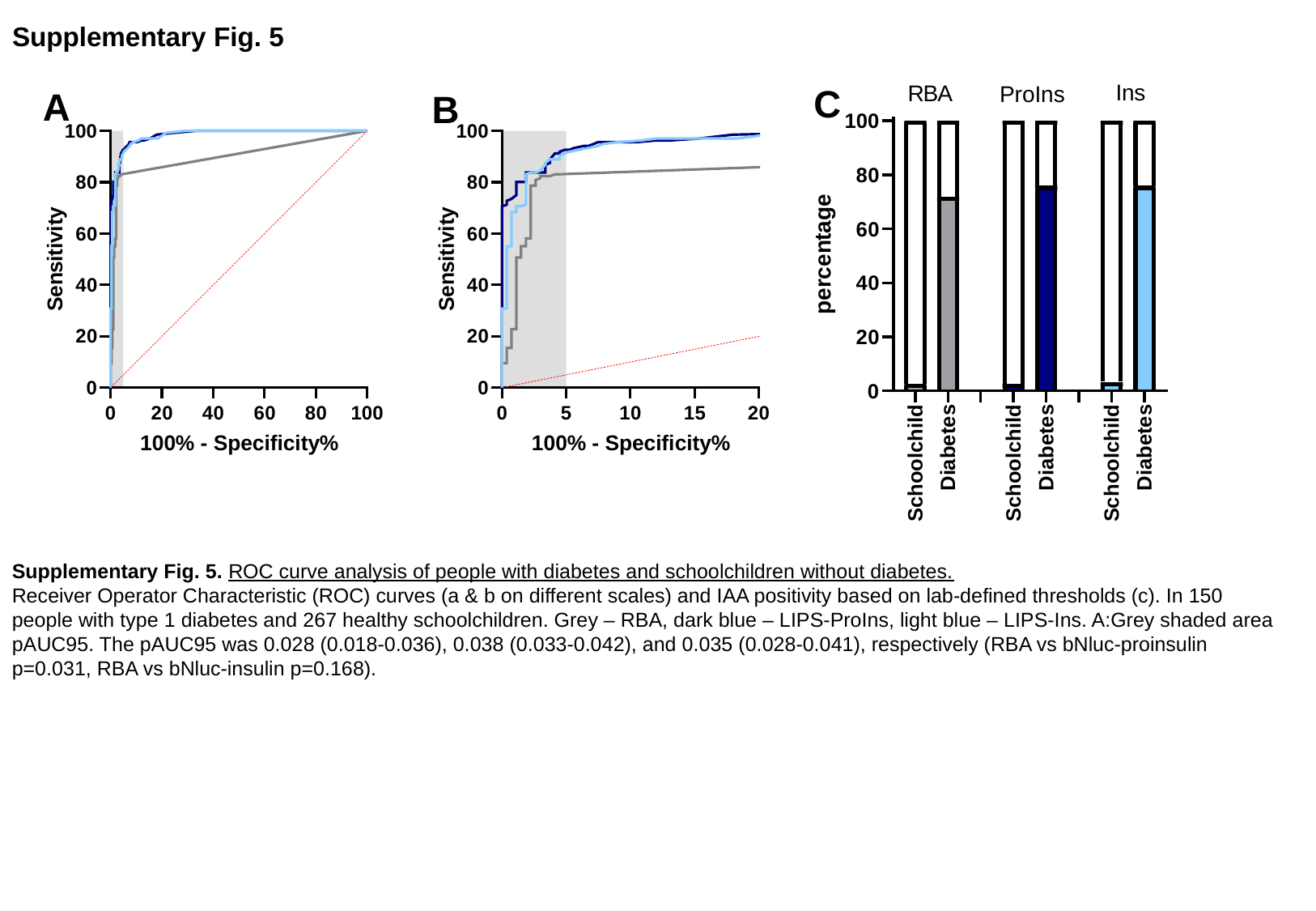

Supplementary Fig. 5
Supplementary Fig. 5. ROC curve analysis of people with diabetes and schoolchildren without diabetes.
Receiver Operator Characteristic (ROC) curves (a & b on different scales) and IAA positivity based on lab-defined thresholds (c). In 150 people with type 1 diabetes and 267 healthy schoolchildren. Grey – RBA, dark blue – LIPS-ProIns, light blue – LIPS-Ins. A:Grey shaded area pAUC95. The pAUC95 was 0.028 (0.018-0.036), 0.038 (0.033-0.042), and 0.035 (0.028-0.041), respectively (RBA vs bNluc-proinsulin p=0.031, RBA vs bNluc-insulin p=0.168).

## Slide 7
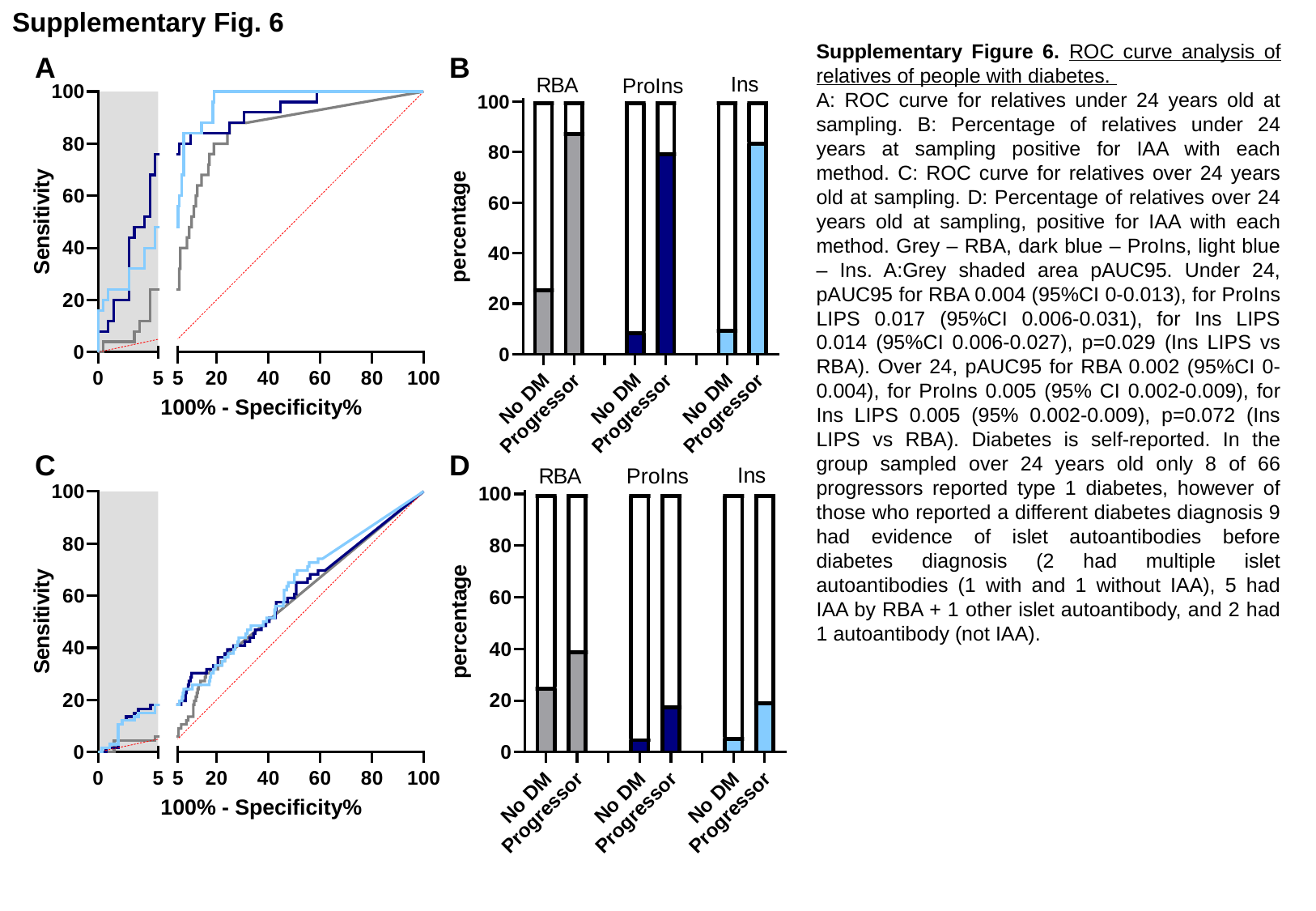

Supplementary Fig. 6
Supplementary Figure 6. ROC curve analysis of relatives of people with diabetes.
A: ROC curve for relatives under 24 years old at sampling. B: Percentage of relatives under 24 years at sampling positive for IAA with each method. C: ROC curve for relatives over 24 years old at sampling. D: Percentage of relatives over 24 years old at sampling, positive for IAA with each method. Grey – RBA, dark blue – ProIns, light blue – Ins. A:Grey shaded area pAUC95. Under 24, pAUC95 for RBA 0.004 (95%CI 0-0.013), for ProIns LIPS 0.017 (95%CI 0.006-0.031), for Ins LIPS 0.014 (95%CI 0.006-0.027), p=0.029 (Ins LIPS vs RBA). Over 24, pAUC95 for RBA 0.002 (95%CI 0-0.004), for ProIns 0.005 (95% CI 0.002-0.009), for Ins LIPS 0.005 (95% 0.002-0.009), p=0.072 (Ins LIPS vs RBA). Diabetes is self-reported. In the group sampled over 24 years old only 8 of 66 progressors reported type 1 diabetes, however of those who reported a different diabetes diagnosis 9 had evidence of islet autoantibodies before diabetes diagnosis (2 had multiple islet autoantibodies (1 with and 1 without IAA), 5 had IAA by RBA + 1 other islet autoantibody, and 2 had 1 autoantibody (not IAA).
